# Emotional arousal when watching drama increases pain threshold and social bonding

**DOI:** 10.1098/rsos.160288

**Published:** 2016-09-21

**Authors:** R. I. M. Dunbar, Ben Teasdale, Jackie Thompson, Felix Budelmann, Sophie Duncan, Evert van Emde Boas, Laurie Maguire

**Affiliations:** 1 Department of Experimental Psychology, University of Oxford, South Parks Road, Oxford OX1 3UD, UK; 2 Calleva Research Centre, Magdalen College, Oxford OX1 4AU, UK; 3 Faculty of Classics, University of Oxford, 66 St Giles, Oxford OX1 3LU, UK; 4 Faculty of English, University of Oxford, Manor Road, Oxford OX1 3UL, UK

**Keywords:** tragedy, pain threshold, endorphins, social bonding, emotional arousal

## Abstract

Fiction, whether in the form of storytelling or plays, has a particular attraction for us: we repeatedly return to it and are willing to invest money and time in doing so. Why this is so is an evolutionary enigma that has been surprisingly underexplored. We hypothesize that emotionally arousing drama, in particular, triggers the same neurobiological mechanism (the endorphin system, reflected in increased pain thresholds) that underpins anthropoid primate and human social bonding. We show that, compared to subjects who watch an emotionally neutral film, subjects who watch an emotionally arousing film have increased pain thresholds and an increased sense of group bonding.

## 1. Introduction

Fiction, in the form of both storytelling and drama, is an important feature of human society, common to all cultures. Though widely studied in the humanities, the reasons why we become so engrossed in fiction, and the likely functions for this, have attracted very little attention from either psychologists or behavioural biologists. Yet, it is evident that people are willing to spend a great deal of time, and often money, to be entertained in this way, whether casually in social contexts or formally in the theatre or cinema, often incurring significant costs when doing so. Storytelling forms a major component of evening conversations around the campfire in hunter–gatherer societies [1]. One important function is that it enables us to pass on, in the form of origin stories or a corpus of commonly held folktales and folk knowledge, the cultural ideologies that create a sense of community. Shared knowledge forms part of the mechanism that binds friends [2–5] as well as communities [6,7].

As important as these cognitive aspects of storytelling may be for community bonding, they do not explain why we are willing to return again and again to be entertained by storytellers and dramatists. One plausible explanation for our enjoyment of comedy might be that comedy makes us laugh, and laughter activates the endorphin system [8–11], thereby providing a sense of reward and pleasure. Endorphins act as analgesics and increase tolerance of pain [12], being responsible for well-known phenomena like the ‘runner's high’ [13]. As a result, comedy that makes us laugh out loud results in an increase in pain threshold [8–10]. But why should we be just as engaged by emotionally stirring plots that ‘reduce us to tears’ (i.e. tragedies)? One possibility is that the emotional arousal triggered by such stories also activates the endorphin system, because the same areas of the brain that support or respond to physical pain are also involved in psychological pain [14–17]. There is now an extensive literature suggesting that social rejection or viewing emotionally valenced pictures, and even just musically induced mood change, elevate pain thresholds, thereby seeming to allow subjects to attenuate their responses to negative emotional experiences [18–22]. There is even some suggestion that watching a dramatic film increases pain threshold, albeit with small samples and somewhat mixed results [23,24].

While the cognitive component of social bonding is important in maintaining relationships through time in humans, primate social relationships and the bonding of social groups in humans, it is also underpinned by a psychopharmacological mechanism in what is effectively a dual mechanism process [25]. Endorphins, while part of the brain's pain management system [12,26–29], also play a central role in social bonding in anthropoid primates [30–33]. This latter effect is mediated through the afferent c-tactile neural system [34] by the light stroking that occurs during social grooming, and PET imaging has confirmed that this behaviour activates the endorphin system in humans [35]. It seems that a number of other social activities, including laughter [8], singing [36] and dancing [37], also activate this system and, through this, enhance the sense of bonding to the other individuals present.

We used live audiences to test the hypothesis that emotionally arousing film drama triggers an endorphin response (indexed by change in pain threshold) and, at the same time, increases the sense of belonging to the group (social bonding).

## 2. Material and methods

We used an emotionally intense made-for-TV film (*Stuart: A Life Backwards*; 90 min), based on a real-life personal story [38]. The film portrays the life story of Stuart, a disabled and homeless child abuse survivor, often in harrowing detail, and provides a disturbing insight into how a disabled child could end up being driven to prison, drugs, hopelessness, a life on the streets and eventual suicide. In all, 169 participants (101 females; mean age = 24.8 ± 10.2 years, range 18–72) watched the film in a small theatre environment in groups of varying size (mean 11.3, range 2–49). As a control condition, 68 participants (42 females; mean age = 29.7 ± 12.3 years) watched two documentaries (*The Museum of Life, Episode One* (BBC, 2010; 60 min) and *Landscape Mysteries: In Search of Irish Gold* (BBC, 2008; 30 min)) in a continuous back-to-back presentation in groups of 12–20 participants (mean = 16.7). Participants were solicited separately for the two series of films with different advertisements, and were not aware of the alternative or the hypothesis being tested. For practical reasons, the experimental series was run first and the control series afterwards.

While we tried to ensure that participants did not watch the film with friends, it was not possible to ensure that they were wholly unfamiliar with the other members of their assigned group. Of the 169 participants in the experimental group, 74 (44%) stated that they did not recognize anyone in their group and only 13 (8%) recognized more than two other people in their group (and all but one of these were in large groups).

After signing a written consent form, participants completed (i) the inclusion-of-other-in-self scale (IOS [39]; where, for present purposes, ‘other’ referred to the group with whom they watched the film, and thus functioned as an index of group-belonging or bonding), (ii) the short PANAS affect scale providing separate indices for positive and negative affect [40], and (iii) a pain threshold assay. Participants then watched the assigned film (for 90 min) and finally repeated all three tasks (IOS, PANAS and pain threshold). The psychological scales were 7-point Likert self-rating scales. In each case, our measure is the change in a variable indexed as after-minus-before. The PANAS affect scale was included both to ensure that participants did (or did not in the control group) experience an affective response in watching the film and to check whether any changes in IOS or pain threshold could be explained by changes in affect alone.

We use the IOS scale as a measure of bonding to, or engagement with, the group that participants watched the film with. The IOS [40] was originally developed to index dyadic relationships: it consists of a series of seven increasingly overlapping pairs of circles, ranging from barely overlapping to almost completely overlapped, and the question: ‘which pair of circles best describes your current relationship with […]?’ While it was originally developed with a specific individual (e.g. romantic partner) in mind, there is nothing intrinsically unique about the conceptual design, and it transfers naturally and intuitively to membership of a group; indeed, in many ways, it captures the essence of what group membership is all about (being merged within a group when one feels part of it) rather better than it captures the essence of dyadic relationships. The IOS has been used to assay bonding to a group in a number of previous studies [36,37,41–43], and it correlates well with other indices of group membership/belonging.

Endorphins do not cross the blood-brain barrier [44–46], and hence can be assayed directly only with difficulty (normally by lumbar puncture or by PET scanning, both of which are stressful and neither of which is practical for live audiences). Because endorphins have strong natural analgesic properties and play a crucial role in the modulation of pain [12,27–30], we followed common practice and used changes in pain threshold as an assay for endorphin activation. As a pain threshold assay, we used a standard skiing exercise, the wall-sit test (also known as the Roman Chair), and measured how long a participant could sit unsupported with the back against a wall [47–49]; the position becomes increasingly painful the longer it is held until the individual can no longer sustain it and falls to the ground. In this respect, the wall-sit test has some advantages over other more conventional pain assays, such as ischaemic pain, cold pressor or algometer, in that it is less open to subjective definitions of what is painful. Participants were always tested in groups of four or five and were retested post-experiment in the same groups. On each occasion, they were reminded that ‘…this is not a competition, but try to hold the pose for as long as you possibly can. Please ignore what everyone else is doing.’ That said, any competition that might arise from being tested in groups can only be beneficial: we really want subjects to hold the position for as long as they possibly can.

One participant chose not to perform the wall-sit task and one participant failed to complete the IOS task after watching the film in the experimental condition. Because of the time required to do the wall-sit tests, we randomly sampled only half of the participants in large groups (47 out of the 92 subjects in groups larger than 20) in the experimental condition. This also helped balance sample sizes across groups of different size. All participants in the control group were sampled.

In the experimental condition, there was no effect of group size on any of the four variables (change in pain threshold, whether indexed as absolute or relative change: *F*_2,120_ = 0.38, *p* = 0.683 and *F*_2,120_ = 0.36, *p* = 0.696, respectively; change in IOS: *F*_2,165_ = 1.41, *p* = 0.248; change in positive PANAS: *F*_2,166_ = 2.21, *p* = 0.113; change in negative PANAS: *F*_2,166_ = 0.09, *p* = 0.916). Nor were the differences in change pain threshold or change in IOS across groups of different size significant in the control condition (pain: *F*_2,65_ = 0.18, *p* = 0.834; IOS: *F*_9,113_ = 0.08, *p* = 0.924). Considering only experimental groups of the same size range (10–20 subjects) as the control condition, the means and variances for the two key dependent variables (change in pain threshold and change in IOS) in these groups were not statistically distinguishable from those in the complete sample (*p* ≥ 0.995 2-tailed). Group size is thus unlikely to be a confound in any of the analyses.

**Figure 1.**
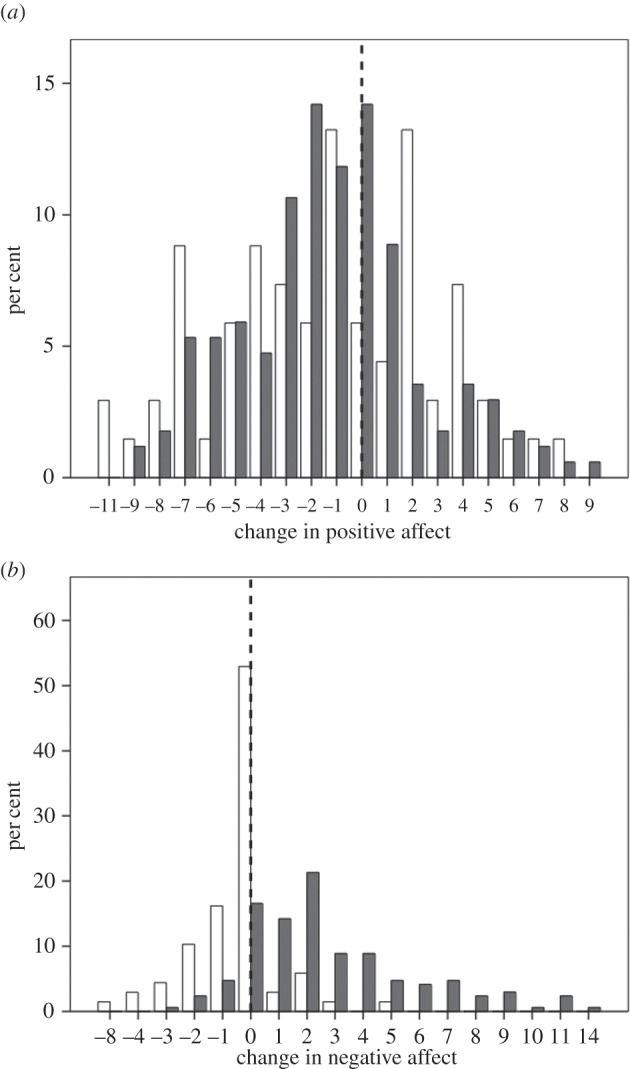
Frequency distribution of (*a*) change in positive affect (after-minus-before composite score) and (*b*) change in negative affect for the experimental (grey bars) and control (white bars) groups. The dashed vertical line indicates 0 (no change).

**Figure 2.**
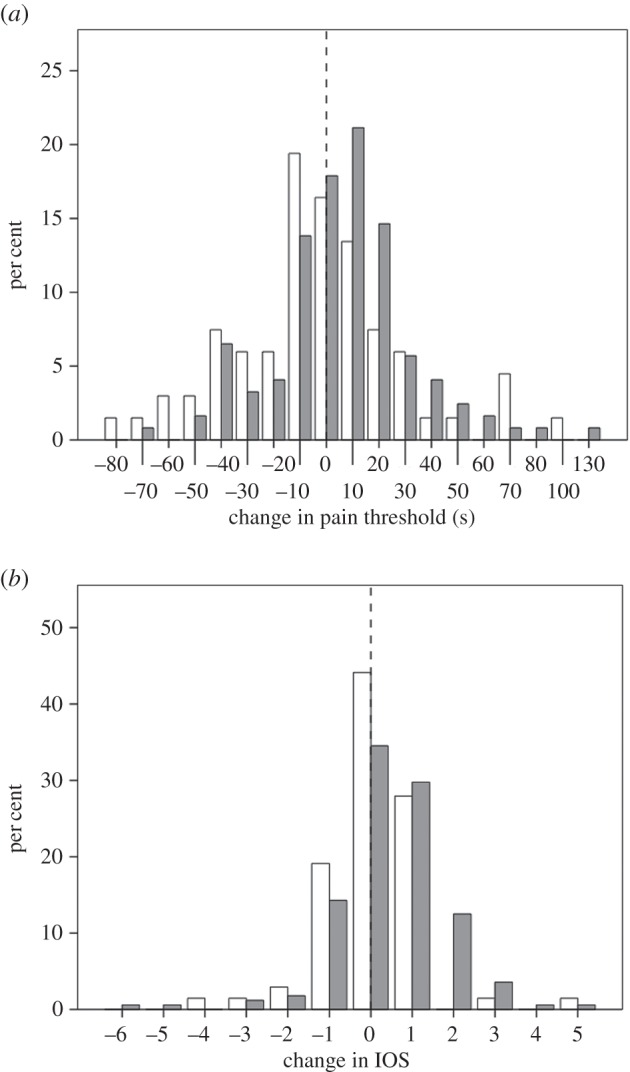
Frequency distribution of (*a*) change in pain threshold (after-minus-before duration for wall-sit task, in seconds) and (*b*) change in IOS rating for experimental (grey bars) and control (white bars) participants. The dashed vertical line indicates 0 (no change).

Although none of the variables differed significantly from normality (Kolmogorov–Smirnov, *p* > 0.05), five individual scores in the experimental condition and two in the control condition were more than 3 s.d. from their respective means on the pain threshold or IOS (figure 2), and, following convention, these values were excluded from all analyses except for the distributional analyses (figures 1 and 2). Note that in the case of the experimental condition our hypothesis is explicitly directional: a significant reduction in pain threshold after watching the film *Stuart* would be at least as strong evidence *against* the endorphin hypothesis as a non-significant result. Where we test for an explicit directional change, 1-tailed statistical tests are appropriate. In all other cases, we use 2-tailed tests. The data are given in the electronic supplementary material.

## 3. Results

As a manipulation check, we first determined whether participants had been emotionally affected by the two films. Watching *Stuart* resulted in a significant reduction in positive affect (figure 1*a*; mean change Δ = −1.36: matched pairs *t*-tests, *t*_128_ = −4.98, *p* < 0.0001) and a very substantial increase in negative affect (figure 1*b*; mean Δ = +2.75: *t*_128_ = −11.66, *p* < 0.00001), whereas watching the control film produced significant, albeit modest, reductions in both positive affect (figure 1*a*; mean Δ = −1.37: *t*_67_ = −2.61, *p* = 0.011) and negative affect (figure 1*b*; mean Δ = −0.47: *t*_67_ = −2.25, *p* = 0.028). We ran mixed models on the two PANAS variables (positive and negative affect), with the condition (*Stuart* versus control films) as a between-subjects effect and change in PANAS score (before to after) and the PANAS × condition interaction as within-subjects effects. There was a significant change in both PANAS scores after watching the films (positive PANAS: *F*_1,234_ = 26.1, *p* < 0.001, *η* = 0.10; negative PANAS: *F*_1,234_ = 31.5, *p* < 0.001, *η* = 0.12), but only in the case of negative PANAS was there an effect of condition (*Stuart* versus control) (positive: *F*_1,234_ = 1.88, *p* = 0.172, *η* = 0.01; negative: *F*_1,234_ = 47.0, *p* < 0.001, *η* = 0.17) or an interaction effect (positive: *F*_1,234_ = 0.02, *p* = 0.887, *η* = 0.00; negative: *F*_1,234_ = 68.1, *p* < 0.001, *η* = 0.23). In sum, to the extent that PANAS indexes affect, responses to the two films differed considerably, with the experimental film *Stuart* inducing a strong negative response, whereas the control film(s) induced only a modest change (most probably due to boredom). The films thus did seem to have the effect we wanted them to have.

Figure 2 compares responses on the two main dependent variables (change in pain threshold from before to after watching the film, and the equivalent change in IOS) for the two films. Neither baseline (pre-film) pain threshold nor baseline IOS differed significantly between the two conditions (pain: *F*_1,188_ = 0.85, *p* = 0.359; IOS: *F*_1,235_ = 0.14, *p* = 0.711). Although not everyone responded with a positive change, the change in both variables was significantly greater than zero for the experimental group (directional tests, paired samples *t*-tests: *p* ≤ 0.010 1-tailed), with no effect due to group size (pain: *F*_2,115_ = 0.99, *p* = 0.374; IOS: *F*_2,160_ = 1.56, *p* = 0.214 2-tailed tests) or sex (pain: *F*_1,115_ = 1.02, *p* = 0.314; IOS: *F*_2,160_ = 0.003, *p* = 0.955 2-tailed tests). By contrast, the control condition did not differ significantly from zero on either measure (*p* ≥ 0.558 2-tailed). The average increase in pain threshold after watching *Stuart* is an increase of +13.1% of the baseline (pre-viewing) threshold. The equivalent value for the control films is a *decrease* of −4.6% of baseline (an effect noted in control groups in previous studies using pain assays [10,33,34]). Since the latter represents what we would expect in the absence of any intervention, this in effect means that the experimental manipulation induced a real net increase in pain threshold of 13.1 + 4.6 = 17.7%.

**Figure 3.**
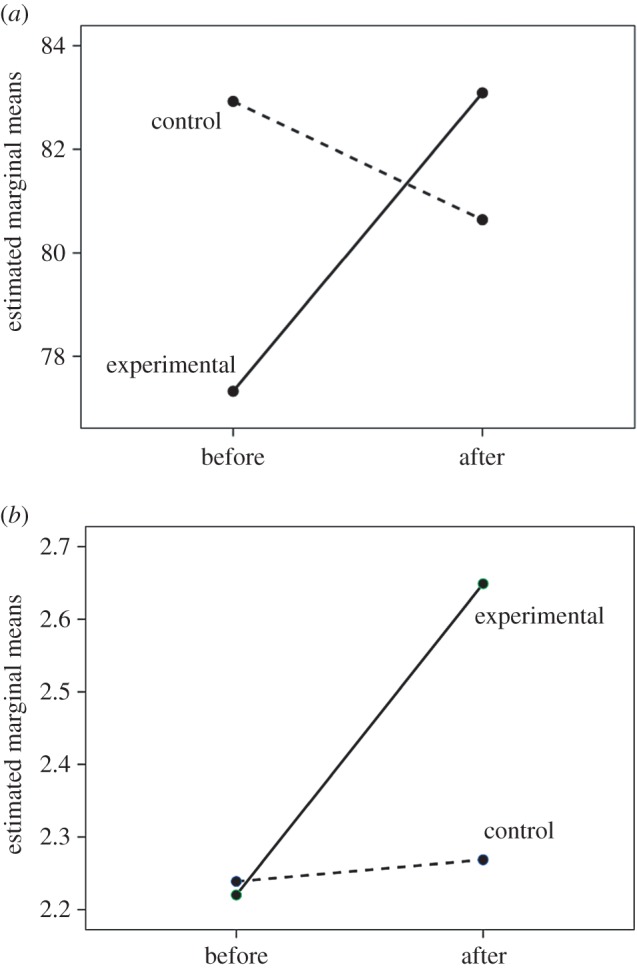
Marginal means for (*a*) pain threshold and (*b*) IOS before and after watching the experimental film (*Stuart: A Life Backwards*) (solid line) or the control films (dashed line).

We used a mixed model GLM to compare responses for the two films directly, with timing (pre- versus post-film, so as to control for pre-film baseline pain threshold) and film type (*Stuart* versus control) as main effects and a directional interaction effect as the crucial test. In respect of pain threshold, there was no main effect of timing (*F*_1,188_ = 0.63, *p* = 0.428) or film (*F*_1,188_ = 0.07, *p* = 0.789) but, importantly, there was a significant timing × film interaction in the predicted direction (*F*_1,188_ = 3.37, *p* = 0.034). The contrast between the two conditions is evident in their marginal means, with pain threshold declining after watching the control film (as is commonly the case [8]) but increasing dramatically in the experimental (*Stuart*) condition (figure 3*a*). For the IOS group-bonding index, there was a significant main effect of timing (in general, IOS was higher after watching any film: *F*_1,233_ = 5.71, *p* = 0.018) but not of film type (*F*_1,233_ = 0.12, *p* = 0.199), and, again, there was a significant timing × film interaction in the predicted direction (*F*_1,233_ = 4.32, *p* = 0.020; figure 2*b*). The marginal means show a strong contrast between the films, with no change in the control condition and a strong positive change in the experimental condition (figure 3*b*).

**Figure 4.**
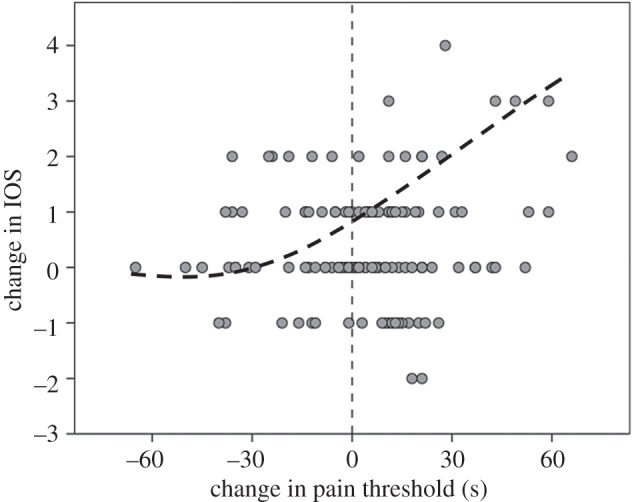
Change in IOS ratings (after-minus-before) plotted against change in pain threshold (after-minus-before duration of wall-sit task, in seconds) for participants in the experimental condition. Five data points more than 3 s.d. of their respective means are excluded. The vertical line indicates no change in pain threshold. The dashed line indicates the separate linear relationships between the two variables below and above Δpain = 0.

**Figure 5.**
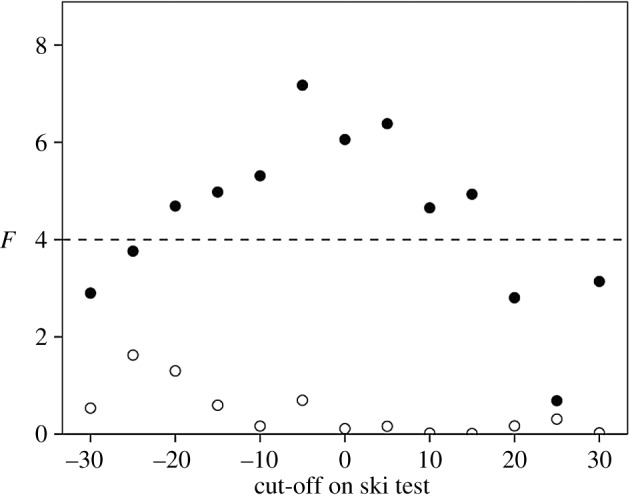
Goodness of fit (indexed by regression *F*-value) for change in IOS regressed on change in pain threshold for data points below (open symbols) and above (solid symbols) a sliding cut-off in pain threshold change for the distribution in figure 4. All values above the horizontal line are statistically significant (*p* < 0.05).

Plotting change in pain threshold against change in IOS for the experimental group (figure 4) yields a distribution that, of the equations available in SPSS, is best fitted within the observed range by a cubic regression with a rising right tail (*F*_3,115_ = 3.35, *r*^2^ = 0.08, *p* = 0.022; best-fit linear equation: *F*_1,117_ = 3.38, *r*^2^ = 0.028, *p* = 0.069), indicating that the relationship is likely to have a nonlinear rather than a linear form. Inspection of the data suggests that the change in IOS is asymptotic on the left-hand side (negative values of Δpain), but increases linearly for positive values of Δpain. Partitioning at successive pain thresholds and fitting separate linear regressions to the left and right halves of the resulting graph suggest that, irrespective of where the break-point is placed, the left-hand side never differs from a slope = 0 (0.226 ≤ *p* ≤ 0.916), whereas the right-hand side has a significantly positive linear fit for all splits between approximately −24** **≤ Δpain ≥ +17 (where 0.009 ≤ *p* ≤ 0.0.036; figure 5). Even with the five outliers on the upper right of the graph removed, the right-hand side relationship is still significant (Δpain > 0: *p* = 0.024). This suggests that not all subjects responded emotionally to the experimental film *Stuart* and that those who did not showed neither an increase in pain threshold nor an increase in bondedness (these individuals behaved much like participants in the control condition); but those who did respond exhibited a linear increase in bondedness that was proportional to their increase in pain threshold.

**Figure 6.**
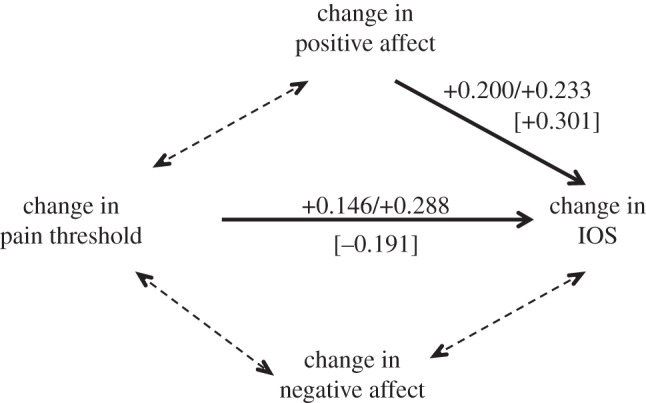
Best-fit model identified by path analysis of the causal relationships between changes in pain threshold and IOS (bondedness) and changes in positive and negative affect. Separate models were tested for the experimental and control conditions. Five subjects in the experimental (*Stuart*) condition and two subjects in the control condition had individual datapoints that were more than 3 s.d. from their respective means, and these individuals were excluded from the analyses. Six increasingly complex models (incorporating the effects indicated by the dashed doubled-headed arrows in different combinations) were compared by maximum likelihood, but the model indicated by the solid arrows provided the best, as well as the most parsimonious, fit. The numbers against the solid arrows are the standardized partial regression coefficients, β, for this best-fit model; the values without brackets are those for the experimental film (*Stuart*) (with the first value being for all data and the second that for Δpain > 0 only), while those in square brackets are the equivalent values for the control condition films. All coefficients except that for pain threshold → IOS in the control condition are significant.

We used path analysis to determine whether this relationship might simply be due to changes in affect. We considered change after watching the film for the four key variables (pain threshold, IOS, and positive and negative affect), with a 1-tailed effect of pain on IOS, and 2-tailed effects in all other cases. We considered six alternative models, all of which included the effect of change in positive affect and pain threshold on IOS (group bondedness). For *Stuart*, a comparison of maximum likelihoods yields a best-fit model in which change in positive affect and change in pain threshold independently influence change in IOS, with no other significant effects (figure 6; Fisher's *C* = 22.003; all other models *C* ≤ 16.145). The values against the solid arrows give the standardized slopes for these two effects. Note that although the standardized *ß* for pain threshold is lower than that for positive affect, the model considers only linear relationships, whereas figure 4 shows that the relationship with IOS is actually nonlinear; considering only Δpain > 0, these coefficients are pain = 0.288 (*p* = 0.007 1-tailed) and positive affect = 0.233 (*p* = 0.044 2-tailed). Changes in negative affect have no influence on either pain threshold or IOS, and neither positive nor negative affect had any influence on pain threshold. Note that positive affect and pain threshold both influence IOS in the same direction: the greater the pain response and the higher the positive affect, the higher is the sense of group bonding. As pain threshold increases and positive affect decreases when exposed to this emotionally intense stimulus (figures 1 and 2), this makes it clear that these two variables are acting independently of each other.

We then ran the path analysis on the control condition data. This yielded essentially the same results (positive affect was significantly correlated with IOS, but positive and negative affect were not correlated with anything else), except that the relationship between pain threshold and IOS dropped out. Indeed, if anything, the relationship between pain threshold and IOS was negative (figure 6). We interpret these results as implying that there is a steady underlying background relationship between positive affect and IOS (feeling positive makes you feel more bonded to whatever group you are with, or *vice versa*), but when the endorphin system is stimulated this adds a significant independent extra boost to IOS ratings. This offers strong confirmation for the claim that (i) affect and pain threshold are unrelated and (ii) pain threshold influences IOS independently of affect. It also suggests that the causal direction is change in pain threshold influencing IOS.

## 4. Discussion

We tested the explicit hypothesis that watching an emotionally arousing film increases the sense of belonging to a group, and that this effect is likely to be mediated by the endorphin system. Our results confirm that both pain threshold and sense of bondedness to the group increase after watching an emotionally arousing film, but not after watching films that have no emotional content. Previous studies have shown that this bonding effect is specific to the group with whom one does a relevant activity, and does not increase the sense of bonding to the wider community, even when that community is familiar [37]. The path analysis allows us to conclude that this relationship is independent of the simultaneous change in affect. The extensive neurophysiological evidence for a close relationship between pain tolerance and endorphin activation [12,26–29], combined with the fact that changes in pain threshold following laughter are also explicitly associated with endorphin activation [11], suggests that the increase in pain threshold can be interpreted as indicating endorphin uptake. We therefore interpret the present results as implying that the increase in pain threshold drives the change in IOS and not the other way around (see also [11]), though it would be desirable to confirm this experimentally, perhaps using PET to confirm endorphin system activation.

**Figure 7.**
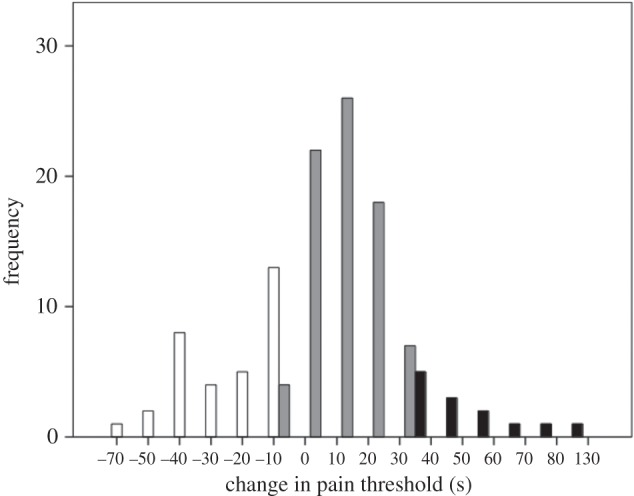
*k*-means cluster analysis of absolute change in pain threshold for the experimental condition suggests an optimal division into three clusters: those who did not respond to the film at all (negative change: white bars), those who responded somewhat (low positive change: grey bars) and those who responded a lot (high positive change: black bars).

Our experiment yields four important results. First, we confirm earlier findings, based on modest samples using short (10 min) film clips with somewhat mixed results [23,24], that watching tragedy results in an increase in pain threshold. Second, while in general there was an increase in pain threshold after watching *Stuart*, not everyone shows this: some people were unmoved by the film. To give some sense of the scale involved, the mean change in pain threshold for those who exhibited a positive change was 20.8 ± 21.3 s (equivalent to an increase of 25% over the mean baseline pre-film value of approx. 79 s), compared to a decrease of −18.5 ± 15.6 s for those exhibiting a negative change. A *k*-means cluster analysis of change in pain threshold suggests an optimal division into three clusters (figure 7) that, respectively, represent those who do not respond at all to the film (Δpain < −5 s), those who respond somewhat (−5 > Δpain < 35 s) and a small subset who respond very strongly (Δpain > 35 s) (with the two sexes evenly split across the three categories: *χ*^2^ = 3.62, d.f. = 2, *p* = 0.623). (All values of *k *≤ 6 tested identify Δpain ≈ 0 as a major division in the data.) Similar results in which some subjects failed to respond have been found in experiments where subjects watched comedy films [10]. Third, for those who did respond, this increase in pain threshold is associated with a heightened sense of belonging to the group (group bonding). Importantly, figure 4 makes it clear that those who do not respond with endorphin activation do not exhibit any change in their sense of group bonding. Fourth, the path analyses of figure 6 suggest that the sense of belonging to the group that is indexed by the IOS is independently influenced by both changes in positive affect and changes in pain threshold (i.e. endorphins). This might imply that what is conventionally labelled as ‘affect’ in the broader psychological literature may in fact consist of two quite different phenomena. Since the PANAS are verbal, it may be that they tap into high-order cognitive reflection that may be unrelated to the more visceral sub-cortical emotional responses that directly trigger an endorphin response.

We have argued that, on balance, our results should be interpreted as a causal sequence in which the emotional response to a film influences pain threshold (interpreted as a signal of endorphin activation), and that this in turn influences the sense of bondedness. There are, of course, two other possible causal models. One is that the causal logic is reversed: watching an emotionally arousing film increases sense of bondedness, and this in turn increases pain threshold. The other is that an emotionally arousing film can influence bondedness indirectly via pain threshold changes as well as directly (although the mechanism for this remains unclear). Although it would be difficult to test experimentally between these alternatives, one possibility (albeit not practical in the current set-up) would be to administer an endorphin agonist such as naltrexone to suppress the endorphin response; if the bondedness was also suppressed, this would allow us to conclude that our suggested causal sequence was the right one. Suppressing bondedness, however, would be more difficult, so running the manipulation in both directions would probably not be possible. A mediation analysis can often be a helpful alternative approach in such cases. Although not ideal given that film type is a dichotomous variable (albeit with an underlying quantitative basis in terms of arousal), a mediation analysis of the data for both films combined does suggest that the causal sequence film type → pain threshold → bondedness (partial effect of film on pain threshold change is significant, whereas the main effect is not: *p* = 0.044 versus 0.068, 2-tailed tests) is more plausible than film type → bondedness → pain threshold (partial effect of film on bondedness is not significant, whereas main effect is: *p* = 0.087 versus 0.045). If the alternative model in which film type influenced bondedness both directly and indirectly turned out to be true, our hypothesis would, of course, still be vindicated.

There remains a question as to whether the greater increase in pain threshold (and hence the putative endorphin effect) in the experimental condition could be attributed to an effect of taking the test in groups, such that individuals become more competitive against each other in the post-viewing test. There are two reasons for thinking this is unlikely. First, for this to explain the results, it would be necessary for the effect to be stronger in the experimental condition than in the control condition. We can think of no principled reason why this might be so, other than the fact that *Stuart* causes a strong emotional response and the control film does not—and that is the point of this study. *How* arousal produces its effect (whether directly or by enabling individuals to be more competitive) is a separate question from the claim we are making (*that* arousal ultimately influences IOS and can do so via the endorphin system). Second, and perhaps more importantly, ours is a within-subjects design, which will reduce the impact of individual differences in competitiveness.

The fact that watching emotionally wrenching drama increases the sense of belonging to the group suggests that our enthusiasm for this form of storytelling might have evolved in the context of bonding social groups. We suggest that this effect probably extends both to listening to stories as well as watching filmed or staged dramas, whenever doing so arouses a strong emotional response. In this respect, storytelling should be seen as supplementing other social mechanisms (laughter, singing and dancing), all of which seem to have very similar endorphin activating properties [9,10,36,37] and all of which enhance group social bonding [36,37,49]. As forms of grooming-at-a-distance, their function seems to be to break through the glass ceiling imposed on social group size by the more conventional one-on-one allogrooming that underpins primate (and even human) social bonding [25,50].

Our focus here has been on one particular aspect of drama. It is not our intention to suggest that all of dramatic fiction can now be reduced to one simple neurochemical. Both the construction of fiction and our enjoyment of it involves many other aspects of human psychology, as well as the more explicitly literary explanatory theories that have emerged within literature studies and linguistics in terms of the ways plots are constructed and language is used. Rather, our concern has been to show that part of the reason we enjoy fiction arises from its ability to influence at least one important neurophysiological system, and that this in turn has implications for how storytelling might be involved in community bonding. If nothing else, our experiment points the way to the potential benefits and insights that collaborations between the humanities and the sciences might have to offer.

## Supplementary Material

Data only

## Data accessibility

The data are provided in the electronic supplementary material.
